# Inadequate Reporting in Randomized Trials: A Silent Threat to Evidence Synthesis

**DOI:** 10.7759/cureus.87690

**Published:** 2025-07-10

**Authors:** Himel Mondal, Seshadri Reddy Varikasuvu, Pradosh Kumar Sarangi

**Affiliations:** 1 Physiology, All India Institute of Medical Sciences, Deoghar, Jharkhand, IND; 2 Biochemistry, All India Institute of Medical Sciences, Deoghar, Jharkhand, IND; 3 Radiodiagnosis, All India Institute of Medical Sciences, Deoghar, Jharkhand, IND

**Keywords:** clinical trial, consort, grade, randomized clinical trial, randomized controlled trial, reporting guideline, systematic review, systematic review and meta-analysis

## Abstract

Systematic reviews and meta-analyses are central to evidence-based medicine, but their validity depends on the quality and completeness of the included randomized controlled trials (RCTs). Despite the existence of guidelines such as the Consolidated Standards of Reporting Trials (CONSORT), inadequate reporting remains a widespread issue, often impeding data extraction and synthesis. Common gaps include the type of RCT, randomization methods, group-wise sample sizes, dropout details, and primary outcome data. Poor reporting threatens the transparency, reproducibility, and utility of clinical research, ultimately compromising the reliability of systematic reviews and the clinical guidelines based on them. To address this, strict adherence to reporting standards must be enforced by journals, reviewers, and funding bodies. Researchers should be encouraged to share tabulated core trial information in public repositories. Educational efforts targeting early-career researchers and stricter accountability mechanisms are crucial. Incomplete reporting must be recognized as a serious obstacle to evidence synthesis and treated as a significant threat to the scientific enterprise.

## Editorial

The growing emphasis on evidence-based medicine has brought systematic reviews and meta-analyses to the forefront of clinical decision-making [1]. Systematic reviews and meta-analyses synthesize data from individual studies to provide clinicians and policymakers with robust, aggregated insights. However, the reliability of such reviews is only as strong as the quality and transparency of the studies they include. A persistent and often overlooked challenge faced by systematic reviewers is the inadequate reporting of essential information in randomized controlled trials (RCTs).

Despite the widespread dissemination of reporting standards such as the Consolidated Standards of Reporting Trials (CONSORT) guidelines [2], many RCTs continue to omit or obscure critical details necessary for accurate appraisal, data extraction, and synthesis. Systematic reviewers frequently encounter studies that fail to clearly define the type of RCT conducted, whether it was parallel, crossover, or cluster-randomized. Similarly, essential elements such as the method of random sequence generation, allocation concealment, and blinding procedures are often missing or vaguely described. Even basic information, such as sample size distribution across groups, dropouts, and reasons for attrition, is inconsistently reported or entirely absent [3]. In the study by Lancee et al. [4], outcome reporting bias was evident in RCTs of antipsychotic drugs, with 85% failing to fully adhere to prespecified outcomes registered on ClinicalTrials.gov. Such discrepancies, particularly in secondary outcomes, raise concerns about the reliability and transparency of published evidence in psychiatric research. Reporting bias can lead to an exaggerated perception of an intervention’s effectiveness and may influence the outcomes of combined analyses in systematic reviews and meta-analyses. Selective reporting and publication bias are more frequently observed in clinical trials funded by the pharmaceutical industry, particularly in fields such as psychiatry, oncology, and cardiology. Trials involving drug interventions, especially those without prior registration or with undisclosed funding sources, are at higher risk for outcome manipulation. Studies from low- and middle-income countries may also face challenges related to transparency and underreporting. These factors collectively contribute to distorted evidence synthesis and may lead to overestimation of treatment efficacy in systematic reviews and meta-analyses.

These deficiencies have serious downstream effects. Missing or unclear information impedes the risk-of-bias assessment, introduces uncertainty into effect size estimations, and may lead to the exclusion of otherwise eligible studies from meta-analyses. When systematic reviewers encounter inadequate reporting in an RCT, they often reach out to the study authors via email to request raw data or clarification on missing or unclear information. However, in many cases, authors do not respond, leaving reviewers with no option but to exclude these ambiguous studies from their analysis. Even when authors do reply, their responses are typically shared only through private correspondence. As a result, the critical information remains unpublished and inaccessible to the wider scientific community, limiting the transparency and reproducibility of the original trial. If another team attempts to include the same study in a future review, they must go through the same process of emailing the authors again, with no guarantee of receiving a response. For example, a pilot test of all eligible Cochrane systematic reviews published in July and August 2015 by Reynders et al. [5] found that 40 of 52 reviews (76.9%) were contacted to obtain additional information for estimating the primary outcome prevalence. This not only hampers efficiency but also undermines the reproducibility and transparency of evidence synthesis.

The causes of poor reporting are multifactorial. Some researchers remain unaware of reporting standards, while others may struggle with space constraints imposed by journals. Additionally, inadequate training in clinical trial design and documentation can lead to poorly structured manuscripts. In rare but concerning instances, selective reporting or intentional omissions may be used to obscure unfavorable results or methodological weaknesses. Regardless of intent, the consequences are substantial. Incomplete reporting erodes the transparency, reproducibility, and reliability of clinical research, ultimately weakening the scientific foundation upon which clinical guidelines and health policy are based. A study by Kaplan et al. reported that knowledge and awareness about reporting guidelines is low: only 13.8% had heard of the Enhancing the QUAlity and Transparency Of health Research (EQUATOR) Network, and just 7% had ever used reporting guidelines in their work. The most recognized guidelines were CONSORT (17.5%), Preferred Reporting Items for Systematic Reviews and Meta-Analyses (PRISMA) (16.3%), and Strengthening the Reporting of Observational Studies in Epidemiology (STROBE) (12.1%). Despite limited knowledge, 82.5% of participants expressed interest in learning more [6]. These findings highlight a significant gap in awareness but also an opportunity. Researchers believe that they need more information and training on reporting guidelines.

**Figure 1:**
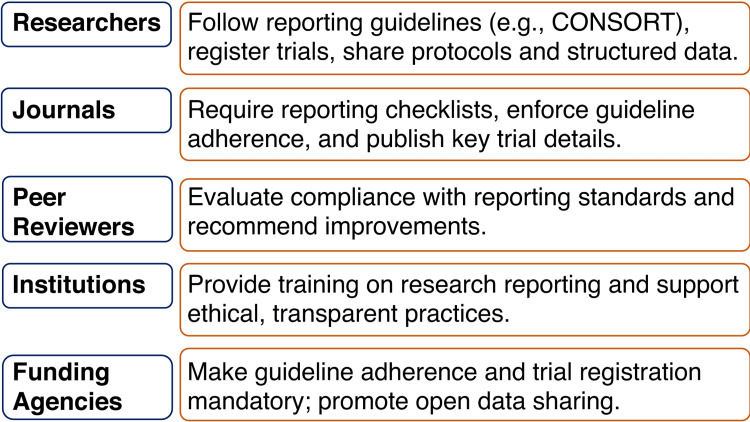
Key stakeholders and their roles in improving clinical trial reporting quality Figure created by Dr. Himel Mondal for this editorial. CONSORT: Consolidated Standards of Reporting Trials.

Improving the current state of poor reporting in clinical trials requires a coordinated, multi-level strategy involving researchers, journals, peer reviewers, institutions, and funding agencies, as summarized in Figure 1. Researchers must be strongly encouraged to adhere to established reporting standards. This adherence should not be limited to the final stages of manuscript preparation; rather, it must be integrated into the research process from the outset, including protocol development, trial registration, implementation, and data analysis. Embedding reporting standards into the workflow promotes consistency, transparency, and accountability at every stage of the research lifecycle.

Equally important is the role of journal editors and peer reviewers in upholding these standards. Editorial boards should take active responsibility for ensuring that submitted manuscripts comply with relevant reporting guidelines. Many journals ask authors to submit a checklist along with the manuscript. This checklist may be published alongside clinical trials for transparency and to facilitate easy identification of information. Reviewers should be trained to evaluate adherence to these guidelines as part of their assessment. In doing so, journals signal that rigorous reporting is not a mere formality but a core aspect of scientific integrity and quality.

Beyond checklist compliance, trial authors can further promote transparency and reproducibility by proactively providing structured data summaries that detail essential methodological and outcome-related information. These structured tables should include specifics such as the type of randomization, allocation concealment procedures, group-wise sample sizes, blinding methods, primary and secondary outcomes, reasons for dropouts, and statistical analysis approaches. Including these as supplementary files allows readers, reviewers, and systematic reviewers to access critical data at a glance, reducing ambiguity and minimizing the need for post-publication author correspondence. To further enhance accessibility, these supplementary materials can be archived in publicly available repositories or linked through trial registries, enabling long-term discoverability and use by future researchers. Table 1 illustrates the types of information most commonly sought by systematic reviewers when evaluating clinical trials [7].

**Table 1:** Major components to mention in a randomized controlled trial CTRI: Clinical Trials Registry–India,  RCT: randomized controlled trial, SD: standard deviation.

Include	To mention
Trial registration number	The trial registration number (e.g., CTRI registration number)
Type of RCT	Parallel, crossover, etc.
Number of arms	Number of groups compared (e.g., 2-arm, 3-arm trial)
Randomization method	How participants were randomized (e.g., computer-generated, block randomization)
Blinding	What type of blinding was used (e.g., single-blind, double-blind)
Allocation concealment	Method used to conceal allocation sequence (e.g., sealed opaque envelopes, central randomization)
Sample size	Number of participants randomized and analyzed in each group
Intervention	Description of the experimental treatment (e.g., type, dose, duration)
Comparator	Description of control group (placebo, standard care, etc.)
Type of analysis	Whether intention-to-treat (ITT), per-protocol (PP), or both were used for analysis
Primary outcome	Outcome names, group-wise values (e.g., mean ± SD), between-group differences, confidence intervals, p-values

Educational initiatives targeting early-career researchers and postgraduate trainees in health sciences should integrate training on reporting standards and evidence synthesis. Understanding how primary studies feed into the evidence pipeline can instill a sense of responsibility toward rigorous and transparent reporting.

The broader biomedical community must acknowledge that systematic reviews are not immune to the flaws of their constituent studies. In fact, they often magnify these flaws if poor-quality data are pooled without scrutiny. Strengthening reporting practices in clinical trials is not merely an academic exercise. It is a necessary safeguard to ensure that clinical decisions, public health strategies, and patient outcomes are grounded in sound evidence. We must move beyond viewing poor reporting as an inconvenience and recognize it as a significant threat to the credibility of scientific evidence. The call to action is clear: better reporting is not optional; it is essential.

Proposed direction for enhancing reporting bias assessment

Currently, most tools used to assess reporting bias in RCTs, such as the Cochrane Risk of Bias tool or the Risk of Bias in Systematic Reviews (ROBIS), rely heavily on qualitative judgments by review authors. While structured, this approach remains inherently subjective and prone to variability. We propose that future efforts could focus on developing a composite, quantitative scoring system with predefined cutoffs to assess reporting bias more objectively. Such a system could potentially inform decisions about the inclusion or exclusion of studies in meta-analyses, thereby encouraging trial authors to adopt more rigorous and transparent reporting practices. Although many journals require an assessment of publication bias, there is still a notable lack of consistency in how this is reported across clinical trials. The introduction of standardized, quantitative metrics may help reduce this variability and strengthen the reliability of evidence synthesis in systematic reviews.
